# Hatching date influences winter habitat occupancy: Examining seasonal interactions across the full annual cycle in a migratory songbird

**DOI:** 10.1002/ece3.7500

**Published:** 2021-06-26

**Authors:** Michael E. Akresh, David I. King, Peter P. Marra

**Affiliations:** ^1^ Department of Environmental Studies Antioch University New England Keene NH USA; ^2^ Department of Environmental Conservation University of Massachusetts Amherst Amherst MA USA; ^3^ U.S. Forest Service Northern Research Station University of Massachusetts Amherst Amherst MA USA; ^4^ Department of Biology and McCourt School of Public Policy Georgetown University Washington DC USA

**Keywords:** carry‐over effects, life‐history trade‐off, parental effects, reproductive cost, stable isotopes

## Abstract

Birds experience a sequence of critical events during their life cycle, and past events can subsequently determine future performance via carry‐over effects. Events during the non‐breeding season may influence breeding season phenology or productivity. Less is understood about how events during the breeding season affect individuals subsequently in their life cycle. Using stable carbon isotopes, we examined carry‐over effects throughout the annual cycle of prairie warblers (*Setophaga discolor*), a declining Nearctic–Neotropical migratory passerine bird. In drier winters, juvenile males that hatched earlier at our study site in Massachusetts, USA, occupied wetter, better‐quality winter habitat in the Caribbean, as indicated by depleted carbon isotope signatures. For juveniles that were sampled again as adults, repeatability in isotope signatures indicated similar winter habitat occupancy across years. Thus, hatching date of juvenile males appears to influence lifetime winter habitat occupancy. For adult males, reproductive success did not carry over to influence winter habitat occupancy. We did not find temporally consecutive “domino” effects across the annual cycle (breeding to wintering to breeding) or interseasonal, intergenerational effects. Our finding that a male's hatching date can have a lasting effect on winter habitat occupancy represents an important contribution to our understanding of seasonal interactions in migratory birds.

## Graphical abstract

Using stable carbon isotopes, we found juvenile male prairie warblers (*Setophaga discolor*) that hatched earlier in Massachusetts, USA, occupied wetter, better‐quality winter habitat in the Caribbean. Repeatability in isotope signatures indicated similar winter habitat occupancy across years; thus, hatching date appears to influence lifetime winter habitat occupancy. We did not find breeding‐to‐wintering carry‐over effects for adult males, temporally consecutive “domino” effects across the annual cycle, or interseasonal, intergenerational effects.

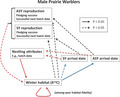

## 1 INTRODUCTION

Migratory animals are affected by events and processes occurring throughout the annual cycle (Faaborg et al., 2010; Harrison et al., 2011). Processes occurring at a given location or time can “carry‐over” to affect an individual at a subsequent location or time (Marra et al., 1998; O'Connor et al., 2014). These carry‐over effects (COEs) are a type of seasonal interaction, and by definition are nonfatal (Marra, Cohen, et al., 2015; Norris & Marra, 2007). COEs have been shown to exist for a wide variety of animals, including insects, amphibians, fish, mammals, and birds (Harrison et al., 2011; Marshall & Morgan, 2011; O'Connor et al., 2014), and documenting these effects is important to better understand fundamental biology and to assist conservation of threatened species (Norris & Marra, 2007).

Studies of migratory songbirds have matched habitat‐specific stable carbon isotopic signatures in tissues of individuals captured at wintering locations with similar signatures from birds captured during the breeding season as a means to examine wintering‐to‐breeding season COEs (Akresh et al., 2019a; Marra et al., 1998; Norris et al., 2004; Rockwell, 2013). On the wintering grounds, isotopic signatures in plants vary along a moisture gradient because of plant photosynthetic pathways, genetic differences, and effects of water stress (Farquhar et al., 1989; McNulty & Swank, 1995; Zhang et al., 1993). Stable carbon isotopes in bird tissues also vary across wet to dry winter habitats as carbon molecules from plants are incorporated into herbivorous insects and then into insectivorous birds (Dawson et al., 2002; Farquhar et al., 1989; Marra et al., 1998). The gradient of moisture and isotopic signatures additionally correlates strongly with winter habitat quality for some migratory passerines, particularly for species wintering in the Caribbean (Akresh et al., 2019b; Latta & Faaborg, 2001; Marra et al., 1998; Smith et al., 2010). Xeric habitats often have less food resources (e.g., insects) for overwintering migrants, especially late in the winter dry season (March–April) (Brown & Sherry, 2006; Parrish & Sherry, 1994; Wunderle et al., 2014). American redstarts (*Setophaga ruticilla*) wintering in dry, poor‐quality habitats have enriched stable carbon isotope values, reduced body condition, and delayed spring departure dates compared with redstarts in wetter, better‐quality habitat (Studds & Marra, 2005, 2007). Redstarts wintering in poor‐quality habitat (i.e., individuals with enriched carbon isotope signatures) have subsequently later arrival dates and lower reproductive success on the breeding grounds, thus exhibiting a wintering‐to‐breeding COE (Norris et al., 2004; Rushing et al., 2016).

Winter rainfall can also interact with habitat quality to impact birds during the winter and subsequently in the breeding period (Akresh et al., 2019a; Studds & Marra, 2007). During a winter with less rainfall, Prairie Warbler (*Setophaga discolor*) body condition and muscle declined at a higher rate in drier compared with wetter habitats in The Bahamas (Akresh et al., 2019b). Yet during a winter with more rainfall, Prairie Warblers maintained body condition across habitat types. Additionally, adult male Prairie Warblers wintering in drier habitats arrive later in the spring on the breeding grounds, but only following specific winters that likely have less rainfall (Akresh et al., 2019a). Similar impacts of winter rainfall have also been observed for other migratory birds (Brown & Sherry, 2006; Robson & Barriocanal, 2011; Rockwell, 2013).

Few studies have examined other potential types of seasonal interactions, such as breeding‐to‐wintering “developmental” or “silver spoon” effects in juvenile migratory birds. Developmental or “silver spoon” effects occur when environmental conditions, processes, or events early in life influence an individual later in life (de Pol et al., 2006; Van Senner et al., 2015), and developmental effects can be classified as COEs (O'Connor et al., 2014). Understanding how developmental processes can impact individuals across seasons is difficult because most migratory songbird populations have low natal survival and site fidelity (<5%) (Schlossberg, 2009) and most songbirds are too small to be tracked with satellite transmitters. Previous studies have shown that juveniles that hatch earlier in the breeding season have more time before fall migration to develop and to increase fat reserves, and early‐hatched juveniles may leave earlier for migration (as shown for savannah sparrows (*Passerculus sandwichensis*) and other species; Morton, 1992, Mitchell et al., 2011, 2012, Meller et al., 2013). Juveniles in better condition as nestlings may also leave earlier for migration (Cam & Aubry, 2011; Merila & Svensson, 1997). Upon arrival to the wintering grounds, earlier‐hatched or better‐conditioned juveniles may be more dominant, and dominant birds can outcompete conspecifics within their age class for better‐quality winter habitat (e.g., in American redstarts and black‐throated blue warblers [*Setophaga caerulescens*]; Wunderle, 1995, Marra, 2000). Birds that arrive earlier have been shown to occupy better‐quality winter habitat in at least one species (Smallwood, 1988). Therefore, in theory (also proposed by Stutchbury, 1994), early‐hatched juveniles that leave for migration earlier and in better condition could arrive on the wintering grounds earlier and in better condition, and may be more likely to acquire and outcompete other juvenile conspecifics for higher‐quality habitat in their first wintering season.

In addition to the effects on juveniles, events such as reproduction during the breeding period may carry over to affect adults in the subsequent non‐breeding season (Briedis et al., 2018; Hoye et al., 2012; Inger et al., 2010; van Wijk et al., 2017). Reproduction can increase stress and affect the timing of molt and migration in migratory passerines, such as in Louisiana waterthrush (*Parkesia motacilla*) and wood thrush (*Hylocichla mustelina*) (Done et al., 2011; Mulvihill et al., 2009; Stutchbury et al., 2011). Adults that fledge young later in the breeding season may molt and depart for migration later (Bogdanova et al., 2011; Fayet et al., 2016; Mitchell et al., 2012; Ogden & Stutchbury, 1996). Later departing individuals may subsequently arrive on the wintering grounds later, as found for a number of migratory passerines carrying geolocators (Cooper et al., 2017; Heckscher et al., 2017; McKinnon et al., 2016), and these individuals may have to settle on poorer wintering habitat due to intraspecific competition (Smallwood, 1988). Thus, there could be a potential life‐history trade‐off between fledging young late in the breeding season versus obtaining high‐quality winter habitat (Inger et al., 2010; Ogden & Stutchbury, 1996). In addition, regardless of the timing of breeding, adults that reproduce successfully may be more stressed and in poor condition at the end of the breeding season compared with unsuccessful adults (Ramos et al., 2018; Stutchbury et al., 2011), and these stressed adults may consequentially be less competitive in acquiring or defending territories in high‐quality winter habitat (Latta et al., 2016). Alternatively, reproductive success and/or timing of breeding may have no effect on winter habitat occupancy, given that adults may have high winter site fidelity and return to the same winter habitats year after year (Johnson et al., 2006; Latta & Faaborg, 2001).

Seasonal interactions may also occur over longer periods than two seasons and accumulate as (a) temporally successive “domino” effects, (b) other long‐lasting, nontemporally successive effects, or (c) intergenerational, interseasonal effects (Catry et al., 2013; Latta et al., 2016; Saino et al., 2017). Specifically, “domino” effects are impacts on an individual that cascade successively across multiple seasons; the term is often used by studies to classify correlations between the timing of departure and arrival schedules of migratory birds at wintering, stopover, and breeding sites (Conklin et al., 2010; Catry et al., 2013; Briedis et al., 2018). Previous studies have found phenological domino effects in relation to avian migration schedules (Gow et al., 2019), and hypothetically, other types of temporally successive effects could also occur across the annual cycle for adults or juveniles. Hatching date could in theory influence wintering habitat quality for juveniles, which could then subsequently affect spring migration schedules or reproductive success on the breeding grounds the following year. Previous studies have shown hatching date can influence spring arrival dates and reproduction in the following year (Gill et al., 2013; Saino et al., 2012; Tilgar et al., 2010), and some studies suggest hatching date can influence an individual's biological “clock” throughout the annual cycle (Both, 2010; Coppack et al., 2001), but few studies have connected these relationships with additional processes on the wintering grounds. Alternatively, instead of a domino effect (breeding to wintering to breeding), the effects on an individual could potentially skip seasons during the annual cycle (Lourenço et al., 2011). For instance, developmental events on the breeding grounds could influence winter habitat occupancy in the subsequent season for a juvenile bird and then continue to impact winter habitat occupancy throughout the individual's life (Johnson et al., 2006; Latta & Faaborg, 2001), but the effect may skip the breeding season if winter habitat does not impact the individual's reproduction (Drake et al., 2013). Other seasonal interactions are also possible, such as intergenerational, interseasonal effects (Senner et al., 2015). For instance, events that impact adults in the non‐breeding season could then subsequently influence their offspring in later seasons.

We conducted a study of seasonal interactions throughout the full life cycle of prairie warblers, a Neotropical–Nearctic migratory songbird that breeds in the eastern United States and winters primarily in the Caribbean (Nolan, 1978). Our present study builds upon previous research on prairie warblers (Akresh et al., 2019b; Latta & Faaborg, 2001), including our previous assessment of wintering‐to‐breeding COEs with the same population (Akresh et al., 2019a). We now examine breeding‐to‐wintering COEs for both juveniles (i.e., developmental effects) and adult males, and long‐lasting, cascading effects across the avian life cycle and across generations. Fledglings in our study population have relatively high natal site fidelity (14%; Akresh et al., 2015), which allowed us the unique opportunity to assess the above relationships.

Specifically, we tested (1) whether nestling hatch date or condition affected the habitat quality occupied by juvenile male and female prairie warblers during the subsequent winter. We used stable carbon isotopes as a signature of winter habitat quality and compared relationships in wet versus dry winters and between sexes (Akresh et al., 2019b; Studds & Marra, 2007). Relationships may differ between sexes because males often occupy taller and wetter vegetation on the wintering grounds compared with females (Akresh et al., 2019b; Latta & Faaborg, 2001); we previously observed this occurs for our Massachusetts population (Akresh et al., 2019a). (2) For adult male prairie warblers, we examined whether reproductive success during the previous breeding season, or hatch date of successful nests, influenced winter habitat quality occupied by the adult. We did not capture sufficient returning, banded females with reproductive data in the previous season to assess impacts on winter habitat occupancy for adult females. (3) We examined year‐to‐year consistency in winter habitat, as inferred from repeated sampling of stable isotopes from adults arriving on the breeding grounds. (4) To investigate interseasonal, intergenerational patterns, we tested whether winter habitat or breeding ground arrival dates of adult males influenced the hatch date of their offspring and then combined this examination with test #1 (if hatch date then influenced the offspring's winter habitat occupancy). Lastly, (5) by combining findings of breeding‐to‐wintering COEs from this study, and wintering‐to‐breeding COEs found previously (Akresh et al., 2019a), we discuss temporally successive domino effects.

## 2 MATERIALS AND METHODS

### 2.1 Study site and focal species

We conducted our study on prairie warblers from 2008 to 2015 at the Montague Plains Wildlife Management Area (MPWMA), in Western Massachusetts, USA (N 42°34′, W 72°31′). The MPWMA is an approximately 600 ha, pitch pine–scrub oak (*Pinus rigida*–*Quercus ilicifolia*) community that is actively managed by the Massachusetts Division of Fisheries and Wildlife. Prairie warblers are small (6–9 g), at‐risk, migratory songbirds that have experienced range‐wide declines over the past few decades (Sauer et al., 2017). Prairie warblers breed in scrub oak thickets, harvested pitch pine stands, and power line corridors in the study site (see Akresh et al., 2015; King et al., 2011) for more details). We studied prairie warblers on nine main plots ranging in area from 4 to 29 ha and also conducted some surveys on adjacent power line corridors to locate dispersing birds (see maps and aerial photographs in Akresh & King, 2016; Akresh et al., 2015, 2017).

### 2.2 Bird surveying and sampling

To quantify arrival dates for male prairie warblers, we surveyed study plots intensively every 2–3 days from 2009 to 2013. Monitoring began in mid‐ to late April prior to the arrival of the first prairie warblers. During each survey, we used handheld global positioning system (GPS) units to record male singing locations. We then utilized these GPS locations to determine male territorial boundaries. From 2009 to 2013, on average, 62% of the arriving males were color‐banded in previous years (range = 45%–82%). We were able to delineate territories for unbanded males prior to capture using GPS locations of these unbanded males (within the matrix of the neighboring banded males) and assumed these unbanded males maintained the same territory before capture (Akresh et al., 2015).

From 2008 to 2015, we captured male and female prairie warblers using a 12‐m mist net, a warbler decoy, and a speaker broadcasting prairie warbler songs. Birds were banded with a United States Geological Survey aluminum band along with a unique color combination of 2–3 colored plastic bands. Using plumage and breeding condition, we sexed birds and aged them into two age classes: either as first‐time breeding individuals that had hatched the previous year and were now in their second calendar year of life (SY), or as after‐second year (ASY) (Pyle, 1997). From 2011 to 2015, we attempted to capture both unbanded and previously banded males within two weeks of their arrival date and sampled the tip (2–3 mm) of the central claw from both feet of all birds for stable isotope analysis (Akresh et al., 2019a).

Claw tips represent dietary isotopic input in the period prior to capture and have a relatively long half‐life (i.e., ~27 days; (Lourenço et al., 2015)). Birds captured soon after arrival on breeding areas should reflect the wintering habitat occupied during the previous winter (Fraser et al., 2008; Hahn et al., 2014). On the wintering grounds, prairie warblers are often territorial and maintain home ranges throughout the winter (Akresh et al., 2019b; Latta & Faaborg, 2001).

To examine isotopic turnover in prairie warbler claws, from 2011 to 2015 we recaptured 13 individual males 10–29 days (mean = 16 days) after their initial capture and sampled additional, different claws (the hallux or the outer claws) than those initially sampled. On average, δ^13^C values in claws from resampled birds were not significantly different from the δ^13^C values of the initial captures (paired *t* test: *t* = 1.3, *p* =.22, mean of the differences = 0.15‰; Appendix S1: Figure S1). Therefore, claw samples from birds captured even several weeks after arrival should represent isotopic input from their winter habitat. Given these findings, we included in our analyses birds that were captured and sampled in May and the first week of June, as these birds were likely captured early enough in the breeding season for the claws to represent the wintering habitat.

We monitored prairie warbler territories and reproductive activity from 2009 to 2013 by visiting each territory every 2–4 days throughout the breeding season. We located nests within territories by conducting systematic searches and observing parental behavior. We assessed the status of the nests every 2–4 days until the nestlings fledged or the contents of the nest had disappeared. When approximately 8 days old, we placed an aluminum band and a unique combination of color bands on nestlings, measured tarsus length with calipers (±0.1 mm), and determined body mass with a digital scale (±0.1 g). We obtained approximate hatch dates by aging young nestlings (0–2 days old) based on their size, amount of down, presence of feather sheaths, and behavior (Nolan, 1978). We defined fledging success within a male's territory as having nestlings present on the eighth day after hatching; nestlings typically fledge when 9–10 days old (Nolan, 1978). We also often confirmed fledging success by observing fledglings outside of the nest, or documenting parents with food during the postfledging period (Martin & Geupel, 1993). If the nest was empty when nestlings were 6–8 days old, we systematically searched for fledglings every 2–3 days to confirm nest failure (Akresh et al., 2015). In 10% of the successful territories, we found adults feeding fledglings, but we were unable to find a nest. In these cases, hatch date could not be determined and these territories were not included in certain analyses. We conducted similar methods from 2014 to 2015, but we surveyed plots and visited most territories once a week, monitored only a smaller subset of territories more frequently to determine reproductive success, and banded fewer nestlings in these years.

### 2.3 Stable carbon isotope analysis

Claws were soaked in a 2:1 chloroform:methanol solution for 2 hr and then dried in a fume hood for 48 hr. We then weighed all samples in tin capsules and combusted them in a continuous flow isotope ratio mass spectrometer (Thermo Scientific Delta V Advantage mass spectrometer coupled with a Costech ECS 4010 elemental analyzer via a ConFlo IV gas interface) at the Stable Isotope Mass Spectrometry Facility at the Smithsonian Institution, Suitland, MD, USA. We ran one in‐house standard for every four unknowns. Stable isotope ratios (^13^C/^12^C) are reported in delta (δ) notation, in per‐mil units (‰) relative to the Vienna Pee Dee Belemnite (δ^13^C) standard. Each sample was unique for claws, although duplicate analyses of prairie warbler blood samples using the same spectrometer were replicable to within 0.2‰ (Akresh et al., 2019a, 2019b).

### 2.4 Statistical analysis

We first tested whether nestling hatch date and condition during the breeding period influenced winter habitat for returning birds that were banded as nestlings. We calculated a size‐corrected nestling body condition index by taking the residuals from a linear relationship of tarsus length regressed over mass (*r* = .58, *t* = 4.2, *p* < .001; McKim‐Louder et al., 2013; Raja‐aho et al., 2017; Evans et al., 2020). The tarsi of nestlings were highly correlated with the tarsi measurements of the same individuals captured as fully grown birds in a following year (*r* = .86, *t* = 9.9, *p* < .001); therefore, tarsus was an appropriate measure of nestling body size (Nolan, 1978). We used δ^13^C values in claws (from returning birds on the breeding grounds) as the response variable to represent winter habitat wetness, and in separate analyses (see below), examined nestling hatch date or body condition. Nestling hatch date and body condition were correlated (*r* = −.46), with later‐hatched nestlings having reduced body condition (*n* = 37, *t* = −3.1, *p* = .004). Thus, we tested hatch date and body condition separately to reduce co‐linearity. There was not enough variation to examine the effects of brood size; 81% of the nestlings were from brood sizes of 3 or 4 (Akresh et al., 2015).

We also hypothesized that a nestling's sex or winter rainfall could interact with hatch date or body condition to influence winter habitat occupancy. The North Atlantic Oscillation (NAO) index is negatively correlated with precipitation in the Caribbean (Jury et al., 2007; Malmgren et al., 1998) and can therefore be used as a measure of winter rainfall. The average monthly NAO index between January and March varied among years (noaa.gov; 2011 = 0.14, 2012 = 0.95, 2013 = −0.57, 2014 = 0.81, 2015 = 1.52), and there were similar NAO values for late winter/March (2011 = 0.61, 2012 = 1.27, 2013 = −1.61, 2014 = 0.80, 2015 = 1.45). Based on the NAO index, we defined 2013 as a wet winter and the other four years as dry winters (Akresh et al., 2019a).

Preliminary analyses showed significant or near‐significant interactions between the effect of our predictor variables (e.g., hatch date) and winter rainfall (based on NAO values) or sex on winter habitat occupancy, so we tested relationships separately for each sex within wet or dry winters. However, sample sizes for some subset groups were low, and we therefore conducted additional tests examining relationships combining across sexes or across years. We conducted mixed‐effects models and included a random effect of year for analyses with five years (i.e., models combining across wet/dry years; Harrison et al., 2018). For the subset models with less than five years, we conducted fixed‐effects models and tested for a fixed effect of year when possible. If the fixed effect of year was not significant, we discarded it from the model to reduce the number of parameters in the final model. We did not include a random effect of individual nest because in our analyses, only five nests had returning siblings from the same nest, and three of these were female–male siblings, which were split when we examined models separately for each sex.

For adult males, we tested whether fledging success (defined as fledging at least one prairie warbler) influenced winter habitat occupancy from 2011 to 2015, using a linear model with δ^13^C values in claws as the response variable and fledging success during the previous year as the predictor variable. For males that fledged young, we also examined the effect of the hatch date of the male's nest on winter habitat occupancy (indicated by δ^13^C values). We used hatching dates in our analyses because nests were checked every few days, and we were uncertain of the exact fledging dates (Saino et al., 2012). We tested effects separately during wet versus dry winters based on the NAO index and included a fixed effect of year and a random effect of individual bird in models examining drier winters. Most (82%) of the returning adult males were after‐third‐year (ATY) when sampling their claws. Given that we found similar results when examining the effects of fledging success on winter habitat for just ATY or third‐year males (age when recaptured), and we had small sample sizes within wet versus dry years, we just presented results on the ages combined. Fledging brown‐headed cowbirds (*Molothrus ater*) may impact male winter habitat occupancy differently than fledging prairie warbler young, but there were no returning males with sampled δ^13^C values that fledged cowbirds during the previous year; cowbird fledging rates in our study site were very low (< 3% of prairie warbler nests; Akresh, 2012). There was not enough variation in the number of prairie warbler young fledged per territory to examine its effect on winter habitat occupancy.

We assessed whether COEs accumulate across the life cycle of prairie warblers by examining temporally successive, domino effects (breeding to wintering to breeding), as well as nontemporally successive effects (breeding to wintering to wintering). To test for nontemporally successive effects, we analyzed the repeatability of winter habitat occupancy for individual birds. We analyzed repeatability (intraclass correlation coefficient) of δ^13^C values in claws from the same individual, for males captured from 2011 to 2015 (Hjernquist et al., 2009; Nakagawa & Schielzeth, 2010). We first conducted a test for all males captured and sampled in multiple years, regardless of age. We then conducted a separate test for males that were captured first as a SY and then later captured as an ASY in a following year (examining just the first year that we caught the individual as an ASY, if the bird was captured multiple times as an ASY).

**FIGURE 1:**
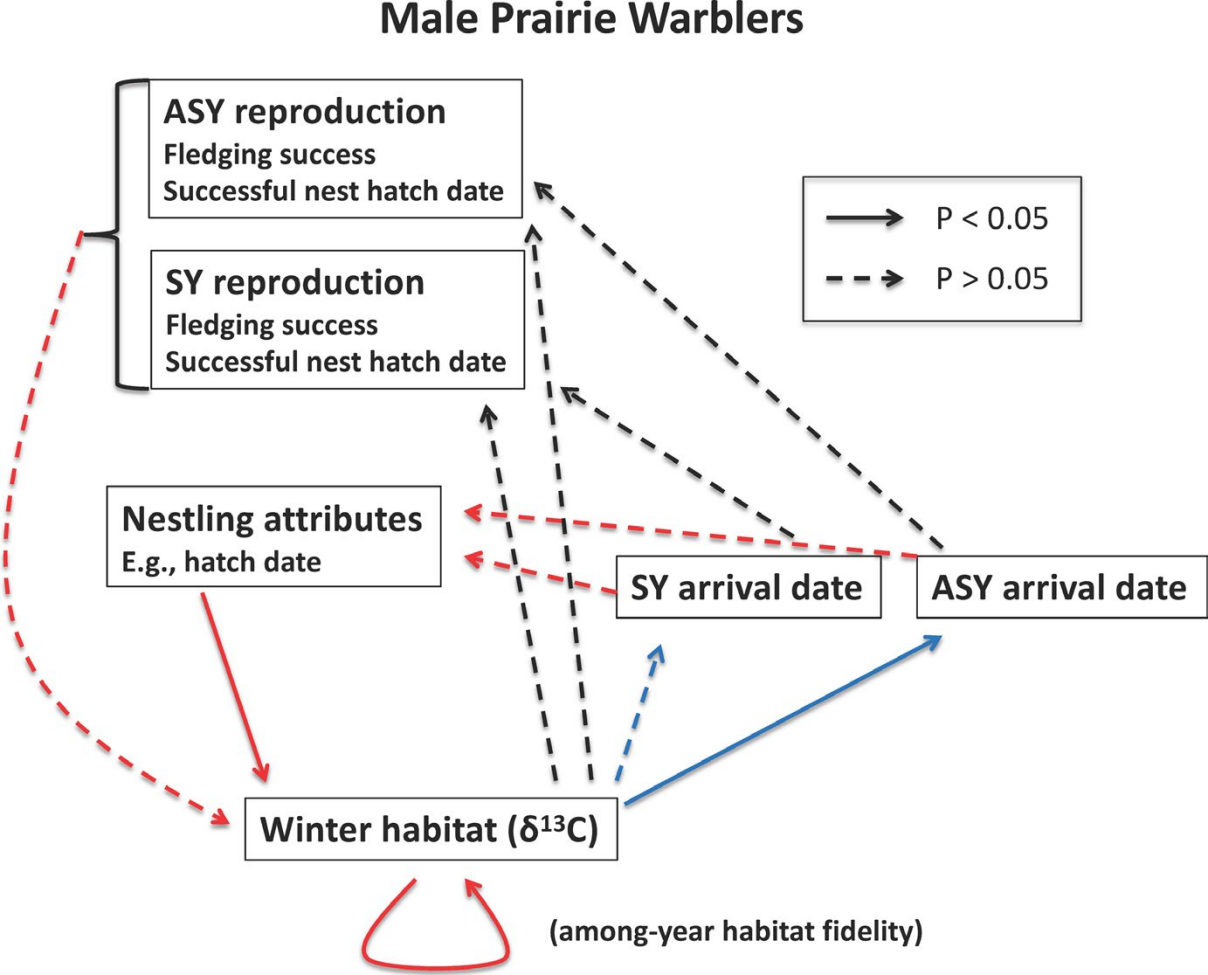
The framework of examined seasonal interactions, and observed results, in a population of prairie warblers breeding in Massachusetts, USA. We combined our present study with a previous study on wintering‐to‐breeding COEs (Akresh et al., 2019a). Blue lines depict results from our previous study, red lines depict results from analyses conducted in this present study, and black lines depict results obtained from both studies. Additional nonsignificant pathways in the breeding season can be viewed in Akresh et al. (2019a). Relationships are shown for just males, examining δ^13^C values in claws to indicate the winter habitat quality that was occupied

To examine interseasonal, intergenerational pathways, we tested whether a male's winter habitat was correlated with the subsequent hatch date of the male's nestlings in successful nests. We also assessed a pathway in which we hypothesized that the winter habitat quality occupied would influence a male's breeding ground arrival date, which would then subsequently influence the hatch date of the male's nestlings (Drake et al., 2013; Norris et al., 2004). Given that we tested the first part of this pathway (winter habitat to arrival date) in a previous study (Akresh et al., 2019a), we only tested the second part of the pathway in our present study (see Figure 1 for a summary of the pathways examined in both studies). Using linear models, we set the hatch date of the male's nestlings as the response variable and, in separate models, tested the effect of δ^13^C values in claws (indicating winter habitat) or male arrival date as the predictor variable. We examined additional relationships within just SY or ASY males, because combining age classes could obscure within age‐class patterns (Drake et al., 2013). Lastly, we examined the effect of winter habitat on hatch date in wet (2013) versus dry (2011 and 2012) winters. We included year and individual as random effects when applicable (e.g., an individual random effect was not possible in models with only SY males). The models with arrival dates used data from 2009 to 2013, while the models with δ^13^C values in claws used data from 2011 to 2013. We only examined hatch dates of nests that successfully fledged at least one prairie warbler young, as we were most interested in determining whether processes would carry over from the adult males to young that were not depredated.

We used the R statistical program version 3.6.2 to conduct all analyses (R Core Team, 2019), and the “lme4” and “lmerTest” packages (Bates et al., 2015; Kuznetsova et al., 2016) to conduct mixed models when appropriate. We analyzed repeatability using the “rptR” package (Nakagawa & Schielzeth, 2010), and we created figures using the “ggplot2” package (Wickham, 2016). We defined significant results as *p* < .05.

## 3 RESULTS

### 3.1 Developmental effects

#### 3.1.1 Hatch date

**FIGURE 2:**
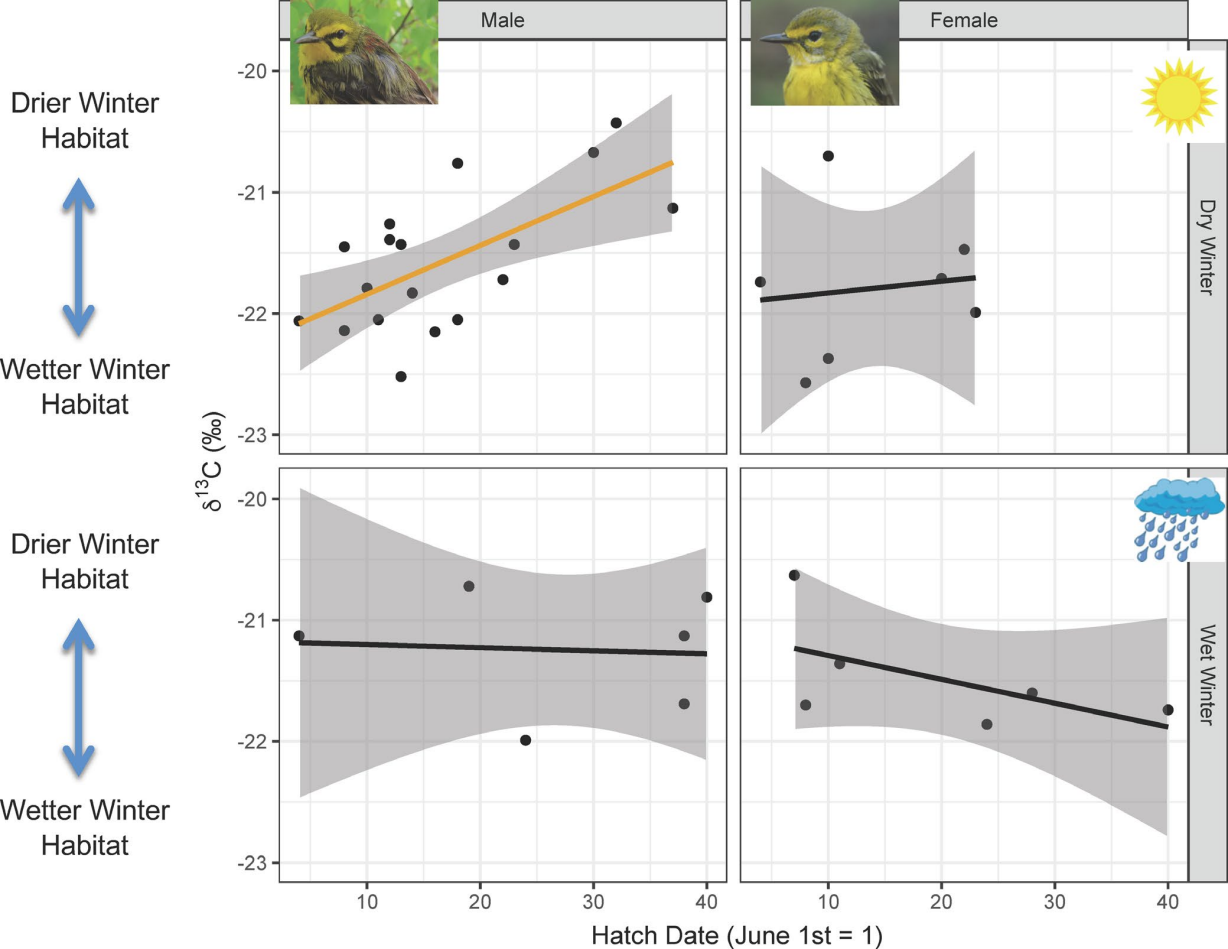
δ^13^C values in SY prairie warbler claws (indicating winter habitat moisture) as a function of nestling hatch date during the previous breeding season. Relationships shown separately for males and females, during drier versus wetter winters based on the mean winter NAO index. Solid lines represent regression lines, while gray shading represents the 95% CI. The orange line denotes a significant relationship, while the black lines denote nonsignificant relationships

Between 2011 and 2015, we captured and obtained stable carbon isotope samples from 24 male and 13 female SY birds that were originally banded as nestlings the previous breeding season. Combining across all years but not across sexes, males that hatched earlier overwintered in wetter, better‐quality habitat (i.e., they had significantly more depleted δ^13^C values; *n* = 24, *t* = 2.5, *p* = .02), compared with males that hatched later. No relationship between hatch date and winter habitat occupancy was found for females (*n* = 13, *t* = −0.6, *p* = .56). However, combining across sexes but not years, during four winters when mean NAO indexes were relatively high (2011, 2012, 2014, and 2015), indicating drier conditions, birds (males and females combined) that hatched earlier had significantly more depleted δ^13^C values (*n* = 25, *t* = 2.9, *p* = .008), but there was no significant relationship during the one wetter winter (2013) when the mean NAO index was low (*n* = 12, *t* = −0.7, *p* = .52). Separating sexes and drier/wetter winters (Figure 2), males had a significant relationship in drier winters between hatch date and δ^13^C values (*n* = 18, *t* = 3.3, *p* = .005); no other relationships were significant (males in the wetter winter, females in dry or wet winters), but sample sizes within these groups were small (*n* = 6 or 7; Appendix S1: Table S1).

#### 3.1.2 Body condition

Combining across all years but not sexes, there was no significant relationship between body condition as a nestling and winter habitat occupancy (δ^13^C values) for males (*n* = 24, *t* = −1.7, *p* = .10), or for females (*n* = 13, *t* = −0.8, *p* = .47). Combining across sexes, there was also no relationship between body condition and winter habitat when the mean winter NAO index was relatively high (dry winters; *n* = 25, *t* = −1.6, *p* = .11), or when the NAO index was low (*n* = 12, *t* = 0.2, *p* = .82). Separating sexes and winters, there were also no significant relationships between body condition and winter habitat occupancy (Appendix S1: Table S1, Figure S2).

### 3.2 Breeding‐to‐wintering COEs in adults

We captured and obtained claw samples from 72 males during 2011–2015 for which we had reproductive success data during the previous breeding season. There was no significant relationship between reproductive success and subsequent winter habitat occupancy (indicated by δ^13^C values) during drier winters (*n* = 55, *t* = −0.7, *p* = .49), or the wetter winter (*n* = 17, *t* = −1.1, *p* = .29; Appendix S1: Figure S3). During the wetter winter (2013), there was a trend in which males that hatched young earlier in the previous breeding season occupied wetter winter habitat (more depleted δ^13^C values in claws), but the relationship was not significant (*n* = 7, *t* = 2.3, *p* = .07). During drier winters, there was no relationship with adult male's hatch date on winter habitat occupancy (*n* = 30, *t* = −0.3, *p* = .78).

### 3.3 Winter habitat fidelity and nontemporally successive effects

**FIGURE 3:**
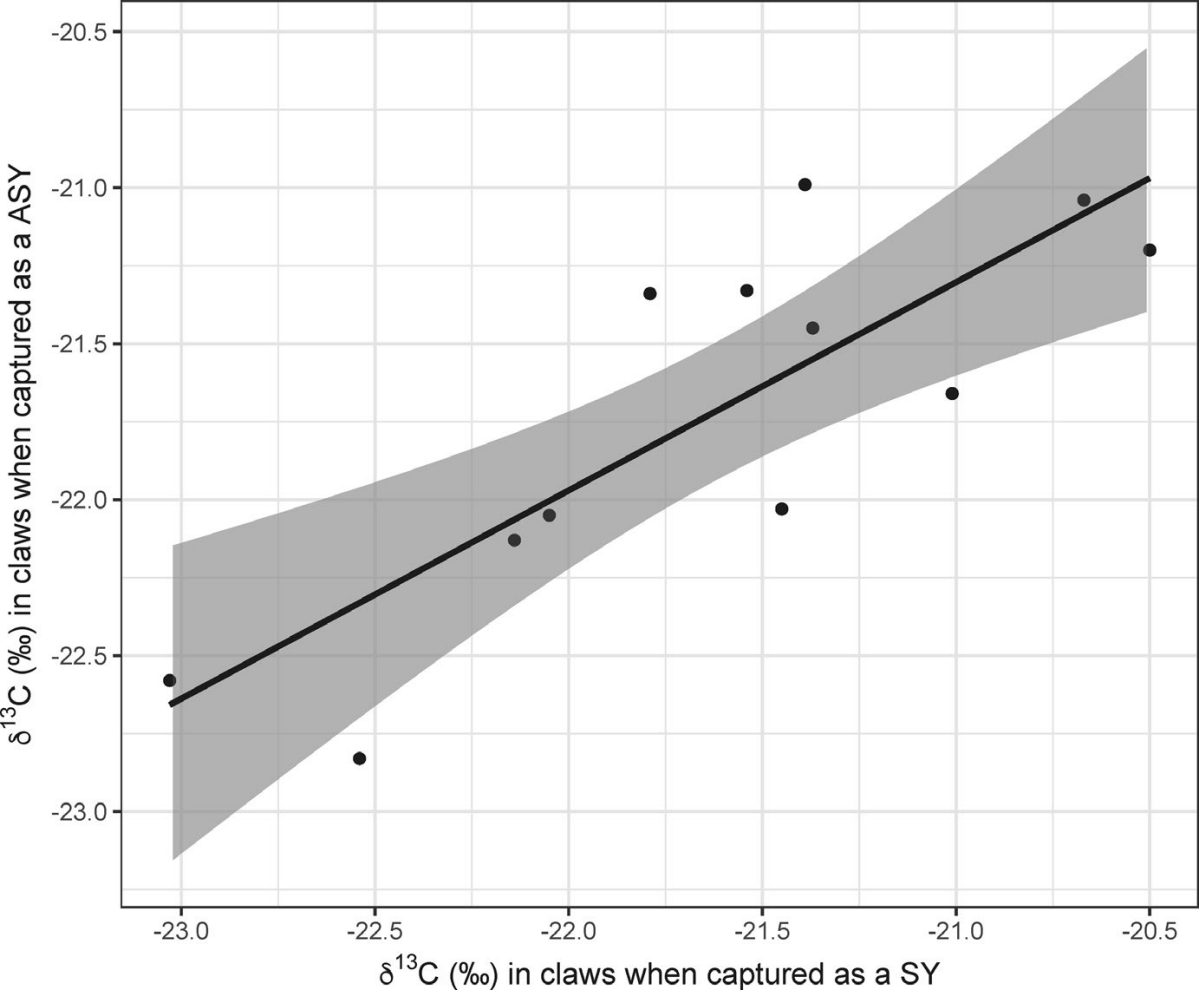
Correlation between the δ^13^C values in claws (indicating winter habitat moisture) of male prairie warblers captured as SY birds and the δ^13^C values in claws of the same individuals captured in a following year. The solid line represents the regression line, while the gray shading represents the 95% CI

The δ^13^C values in claws were highly repeatable among years for the same individual male (*n* = 41 birds caught 92 times, *R* = 0.74, 95% CI = 0.58 to 0.85, *p* < .001), indicating fidelity to winter home ranges or habitats over multiple years. For males that were captured as SYs, and then recaptured in a following year as ASYs, the δ^13^C values in claws were still highly repeatable (*n* = 12, *R* = 0.81, 95% CI = 0.47 to 0.94, *p* < .001, Figure 3). Four of these latter males were banded as nestlings, and these natal returns had small differences in δ^13^C values between years (0, 0.01, 0.37, and 0.40‰; see Figure 3 for comparison). Lastly, three females were captured and then recaptured in a following year (two of which were first captured as a SY); the differences in δ^13^C values between samples were also small (0.14, 0.18, and 0.35‰). Therefore, at least for males, there was some evidence of a long‐lasting effect that was not temporally successive; hatch date affected winter habitat occupancy, which was then consistent over multiple years for the same individual.

### 3.4 Interseasonal, intergenerational effects and domino effects

There were no significant interseasonal, intergenerational effects or domino effects (Figure 1). Habitat occupied by males during the previous wintering season did not affect the hatch date of the successful nests of males, when ages were combined or separated, or during drier or wetter winters across age classes (Appendix S1: Table S1). Additionally, no significant relationship between male arrival dates and hatch dates of successful nests was found across both age classes, or for either age class separated (Appendix S1: Table S1).

## 4 DISCUSSION

Our study provides evidence that developmental events such as hatch date carry over to influence the winter habitat occupied by a migratory bird. Specifically, in four dry winters when NAO indexes were high, earlier‐hatched males occupied wetter winter habitats. Occupying high‐quality, wetter winter habitat is important because Nearctic–Neotropical migratory passerines residing in poor‐quality, drier habitats are more likely to have worse body condition during the winter dry season (Akresh et al., 2019b; Latta & Faaborg, 2001; Smith et al., 2010), which can then influence survival, departure schedules, and reproduction in some populations (Johnson et al., 2006; Latta et al., 2016; Marra & Holmes, 2001; Norris et al., 2004; Studds & Marra, 2007).

Our findings of breeding‐to‐wintering COEs in juvenile males, combined with evidence of high winter habitat fidelity, suggest the effect of hatching date on winter habitat occupancy persists throughout a bird's lifetime. Although few other studies have examined COEs on winter habitat occupancy, other studies have observed that nestling development can affect natal dispersal and breeding habitat selection, which can then influence lifetime reproductive success (Verhulst et al., 1997, Van De Pol et al., 2006, Tilgar et al., 2010). Furthermore, nestlings that fledge earlier, or those in better condition, often have higher survival in their first year of life (McKim‐Louder et al., 2013; Mitchell et al., 2011; Raja‐Aho et al., 2017; Tarof et al., 2011). Given that winter habitat and moisture can influence survival during spring migration (Johnson et al., 2006; Latta et al., 2016; Rockwell et al., 2017), higher annual survival of early‐hatched nestlings in some migratory passerines could potentially be the result of occupying better‐quality winter habitat.

Our results indicate that male prairie warblers occupied winter habitats of similar moisture levels over multiple years and most individuals do not “upgrade” into better‐quality, wetter winter habitat when they become older. Several studies of American redstarts on the wintering grounds, including experimental work, demonstrate redstarts tend to upgrade into better‐quality winter habitat if vacancies become available (Marra, 2000; Studds & Marra, 2005). However, American redstarts, prairie warblers, and several other species of migratory passerines have relatively high annual survival and winter site fidelity (Akresh et al., 2015; Johnson et al., 2006; Latta & Faaborg, 2001; Marra & Holmes, 2001; Nolan, 1978; Wunderle, 1995); thus, there is generally low winter site turnover in these species and winter habitat quality upgrades may only occur for a small proportion of the population. We also previously observed no difference in winter habitat moisture between ASY and SY males in our breeding population (despite a difference between sexes; Akresh et al., 2019a). Given this latter result and the high site fidelity of adults, perhaps most unoccupied winter habitat due to mortality of the previous owner is instead acquired by juvenile birds, with the earlier‐fledging males occupying the higher‐quality vacated sites.

We did not observe any trends or significant effects of hatch date on winter habitat for males during the wetter winter (i.e., in 2013), or for females in either wet or dry winters, but our sample sizes were small for these groups. Perhaps COEs of hatch date are not as important during wetter winters because food availability can be higher across all winter habitat types in these years (Akresh et al., 2019b; Brown & Sherry, 2006; Studds & Marra, 2007), and juvenile birds may have reduced pressure to compete for and maintain territories in wetter habitats during winters with more rainfall. Alternatively, other unexamined factors (e.g., population density on the wintering grounds; Marra, Studds, et al., 2015) may have mediated the relationship between hatch date and winter habitat occupancy in 2013. Our observed differences between sexes in COEs on juvenile winter habitat occupancy may have been due to divergent within‐sex behavioral interactions on the wintering grounds, with males potentially exhibiting a greater degree of territorial behavior and home range exclusion of other same‐sex conspecifics (Cooper et al., 2021; Wunderle, 1995). More territoriality in males compared with females may have led to greater segregation of winter habitat between early versus late‐hatched males, but not in females. Nevertheless, the small sample sizes of males in the wetter year, and the small sample sizes of female natal returns, only half of which (54%, *n* = 7) were captured following drier winters, prevent us from making any definitive conclusions on differences between wet versus dry winters and between sexes.

We observed hatching date had a stronger relationship with winter habitat occupancy compared with nestling body condition, giving some support to the hypothesis that timing constraints could be more important than nestling body condition in influencing subsequent life cycle events (Mitchell et al., 2012, Meller et al., 2013, Åkesson et al., 2017, but see Evans et al., 2020). Potentially, earlier‐hatching juveniles departed earlier for migration (Meller et al., 2013; Mitchell et al., 2012), arrived earlier to the wintering grounds (Cooper et al., 2017), and then obtained higher‐quality winter habitat. However, an alternative mechanism could be the “relative age effect” (Helsen et al., 2005), in which early‐hatched nestlings increased their condition and agility, or improved other beneficial characteristics, by the start of the wintering period compared with late‐hatched nestlings. Early‐hatched nestlings may thus have had a competitive advantage in securing wetter winter habitat compared with later‐hatched nestlings, regardless of their arrival time to the wintering grounds. Both of the above two hypotheses are dependent on strong migratory connectivity, in which prairie warbler nestlings hatching earlier on average in the southern United States (Nolan, 1978) are not competing for the same winter habitat as nestlings hatching later in more northern regions. There is indeed some evidence for strong migratory connectivity in prairie warblers (M. E. Akresh unpublished data, Hobson et al., 2014), although more research is needed. Unfortunately, we did not capture or track juveniles before fall migration or on arrival to the wintering grounds to truly disentangle the mechanisms and determine whether individual quality, migratory timing, body condition before or after fall migration, or intraspecific interactions after arrival on the wintering sites led early‐hatching juveniles to occupy better‐quality winter habitat compared with late‐hatching birds. The stable carbon isotope signatures that we obtained from birds on the breeding grounds only indicate the habitat occupancy during the late winter. We could not determine winter habitat occupancy during the early‐ or mid‐winter periods (Marra, 2000), although late winter habitat occupancy and conditions are more important for subsequent impacts on survival and reproduction (Johnson et al., 2006; Rockwell, 2013).

For returning adult males, we found no association between fledging young during the previous breeding season and winter habitat occupancy. Our finding that successfully breeding males did not obtain poorer‐quality winter habitat conflicts with studies reporting successful reproduction can negatively carry over to affect birds in a following season (Bogdanova et al., 2011; Heckscher et al., 2017; Hoye et al., 2012; Inger et al., 2010; Latta et al., 2016). Perhaps prairie warbler males that are unsuccessful in breeding do not depart for migration earlier. Instead, males may stay on the breeding grounds in early autumn to defend their territories for the following breeding season (as observed for a prairie warbler population in Indiana (Nolan, 1978), and documented for other migrants (Mills, 2005)), mediating any potential COEs on winter habitat occupancy. Additionally, we found adult males returning to previous winter home ranges have high annual habitat fidelity, and therefore, winter habitat occupancy may not be dependent upon the adult male's condition or arrival time to the wintering grounds (Latta & Faaborg, 2001; Marra, 2000). The qualities that enable some males to successfully reproduce may also enable them outcompete conspecifics on the wintering grounds (Byers et al., 2016; Gunnarsson et al., 2005).

We found little evidence for temporally connected domino effects across the annual cycle or intergenerational, interseasonal effects. Combining results from this study with our previous study (Akresh et al., 2019a), hatch date for juvenile males influenced winter habitat, but winter habitat did not subsequently affect arrival date, breeding success, or hatch date of successful nests for SY males in the following year. For adults, although we previously found an association between winter habitat and arrival date on the breeding grounds, there was no effect on reproductive success or hatch dates of successful nests, and there were no breeding‐to‐wintering COEs. Distinctive characteristics of our breeding season study site may have decoupled relationships between winter habitat and reproductive success. High nest depredation rates, especially during the beginning of the season when leaf‐out can be delayed in our study system (Akresh, 2012; Akresh et al., 2015), may have caused decoupling among arrival dates, nest initiation dates, hatch dates of successful nests, and seasonal fecundity (Akresh et al., 2019a; Shustack & Rodewald, 2011). Further research is needed on how winter habitat and arrival date may influence extra‐pair paternity in our study population (Reudink et al., 2009). Nonetheless, our results suggest that some potential seasonal interactions may not occur or accumulate across the annual cycle or across generations because of other overriding environmental factors (Drake et al., 2014; Ockendon et al., 2013; Senner et al., 2014).

## 5 CONCLUSION

Our study provides important evidence for how developmental effects during the breeding season influence migratory birds during subsequent stages of the annual cycle, and increases our understanding of seasonal interactions in migratory animals. For threatened shrubland birds that winter in the Caribbean such as the prairie warbler, maintaining and protecting both high‐quality shrubland breeding habitat and wetter wintering areas may prevent further population declines (Akresh et al., 2015, 2019b; Latta & Faaborg, 2001), but complete annual cycle models incorporating COEs and survival are needed to fully determine the best conservation and management strategies. Moreover, further comprehending effects of climate change should be a priority for future research, as droughts in the Caribbean wintering grounds are expected to intensify (Neelin et al., 2006), and COEs may primarily occur during these dry winters (Akresh et al., 2019a, this study). Future studies that elucidate the potential mechanism for why hatching date influences winter habitat occupancy would also be useful. Overall, understanding how events and environmental conditions throughout the full life cycle interact is essential to determine the limiting factors driving the decline of populations of migratory birds (Norris & Marra, 2007).

## CONFLICT OF INTEREST

The authors have no conflict of interest to declare.

## AUTHOR CONTRIBUTION

**Michael E. Akresh:** Conceptualization (lead); Data curation (lead); Formal analysis (lead); Funding acquisition (supporting); Investigation (lead); Methodology (lead); Project administration (supporting); Resources (supporting); Software (lead); Supervision (supporting); Validation (lead); Visualization (lead); Writing‐original draft (lead); Writing‐review & editing (lead). **David I. King:** Conceptualization (supporting); Funding acquisition (lead); Investigation (supporting); Methodology (supporting); Project administration (lead); Resources (lead); Supervision (lead); Writing‐review & editing (equal). **Peter P. Marra:** Conceptualization (supporting); Data curation (supporting); Funding acquisition (supporting); Methodology (supporting); Project administration (supporting); Resources (supporting); Supervision (equal); Writing‐review & editing (equal).

## Supporting information

Figure S1

Figure S2

Figure S3

Figure S4

Appendix S1

## Data Availability

Data are available from the Dryad Digital Repository: https://doi.org/10.5061/dryad.tx95x69xc.
