# Supplementary figures and images for: Hatching date influences winter habitat occupancy: Examining seasonal interactions across the full annual cycle in a migratory songbird
Source: Ecol Evol. 2021 Jun 26;11(14):9241–53. doi: 10.1002/ece3.7500 (PMC8293775; doi:10.1002/ece3.7500)

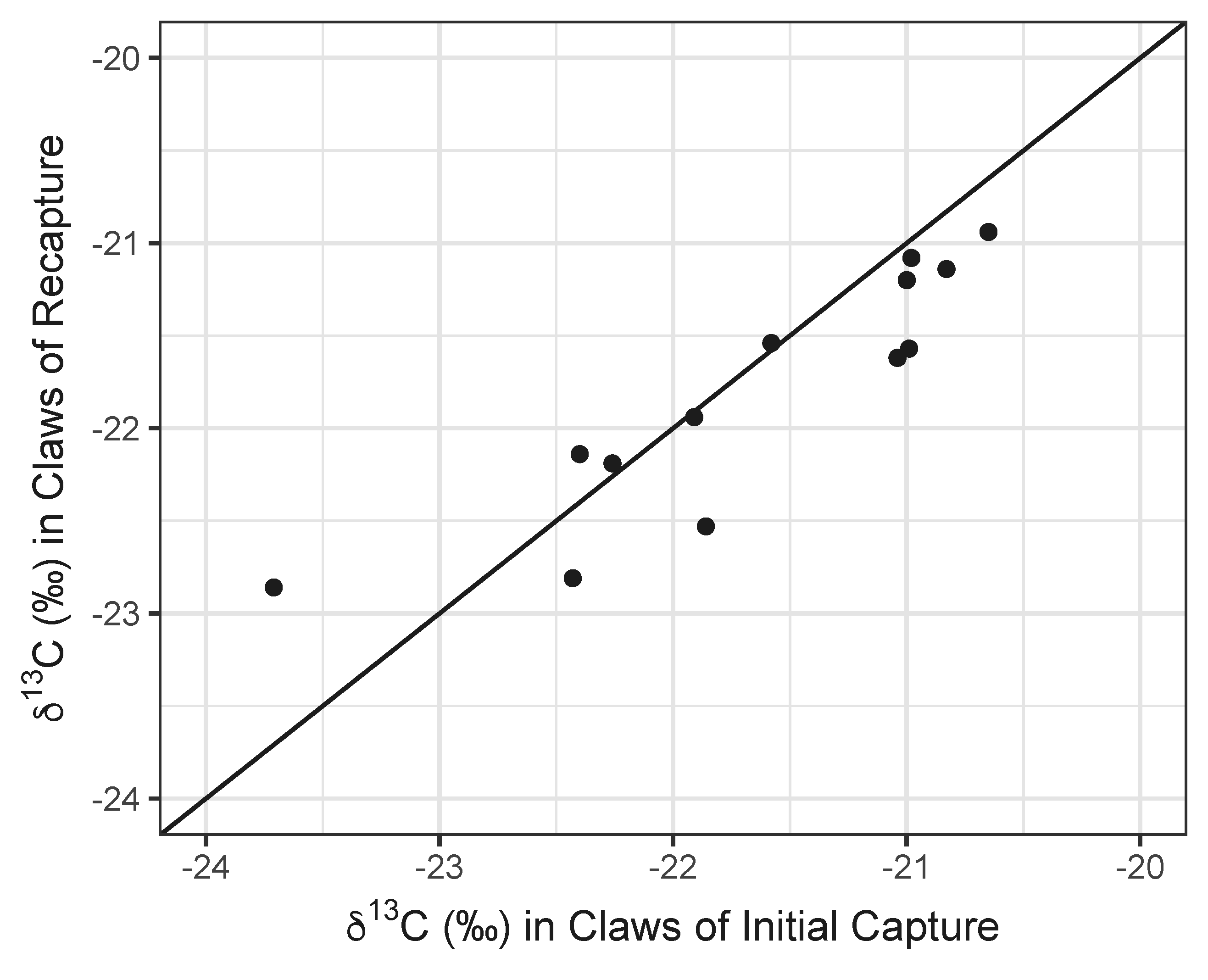

Supplement: Supplementary file 1 — Figure S1 [file ECE3-11-9241-s005.tiff]

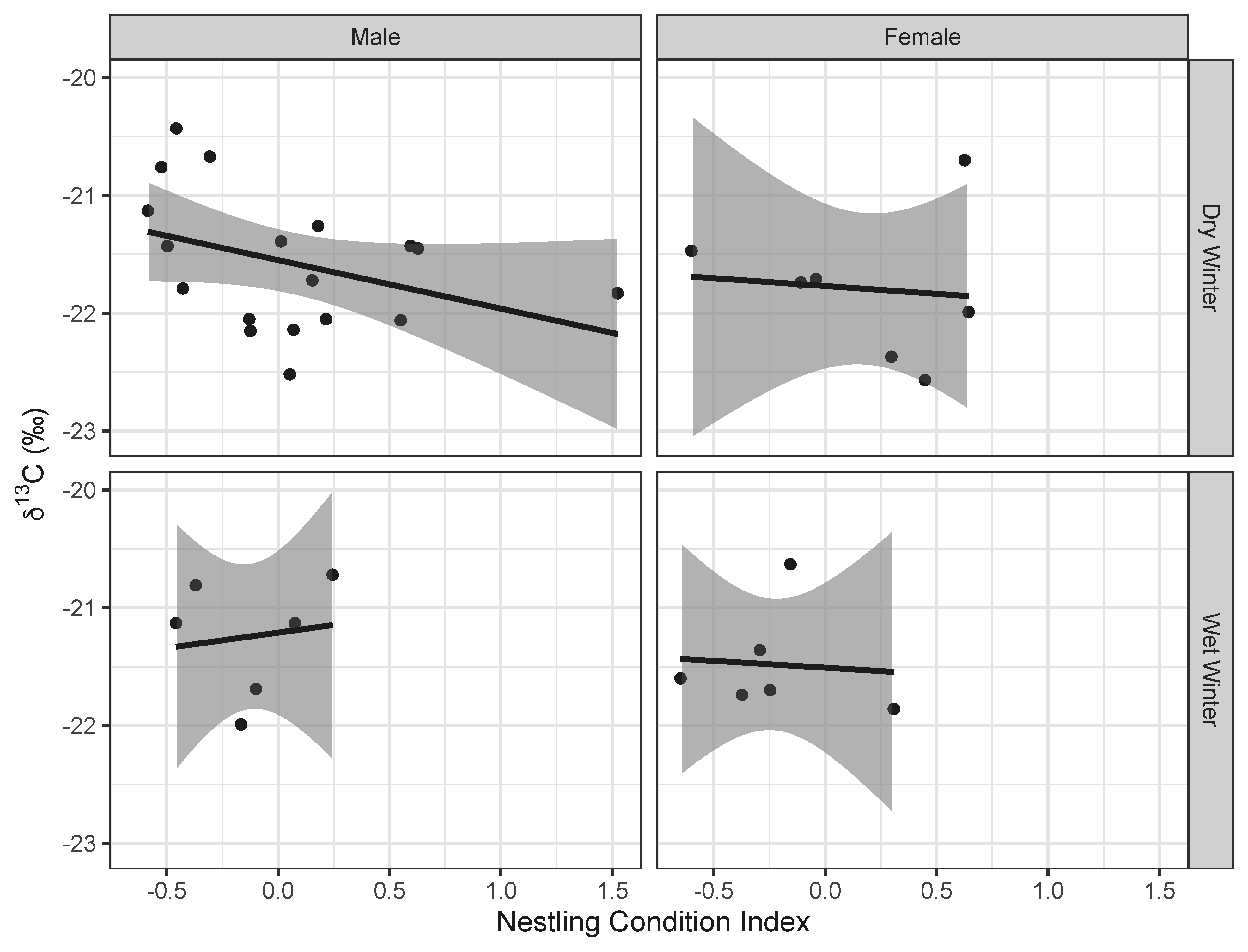

Supplement: Supplementary file 2 — Figure S2 [file ECE3-11-9241-s004.tiff]

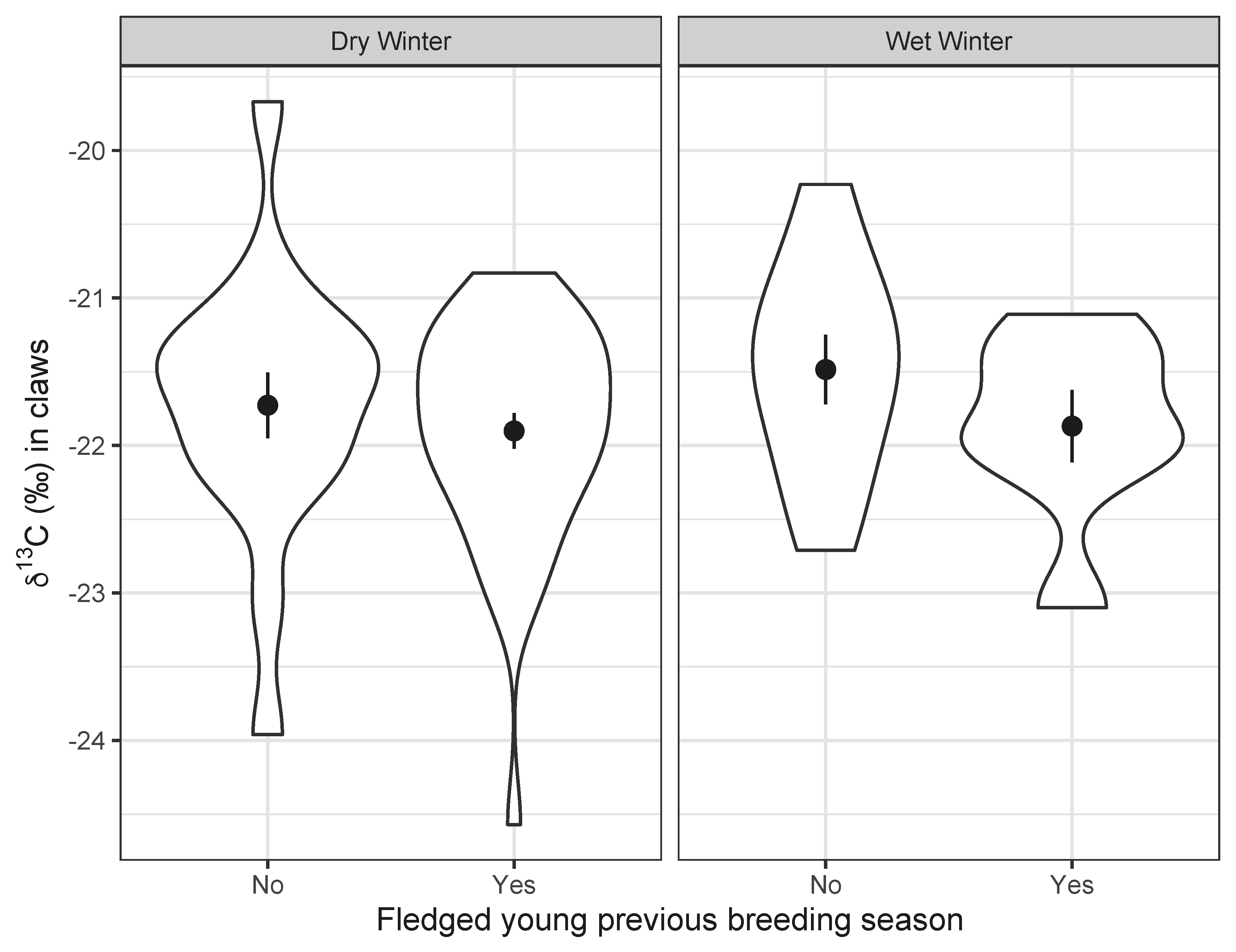

Supplement: Supplementary file 3 — Figure S3 [file ECE3-11-9241-s001.tiff]

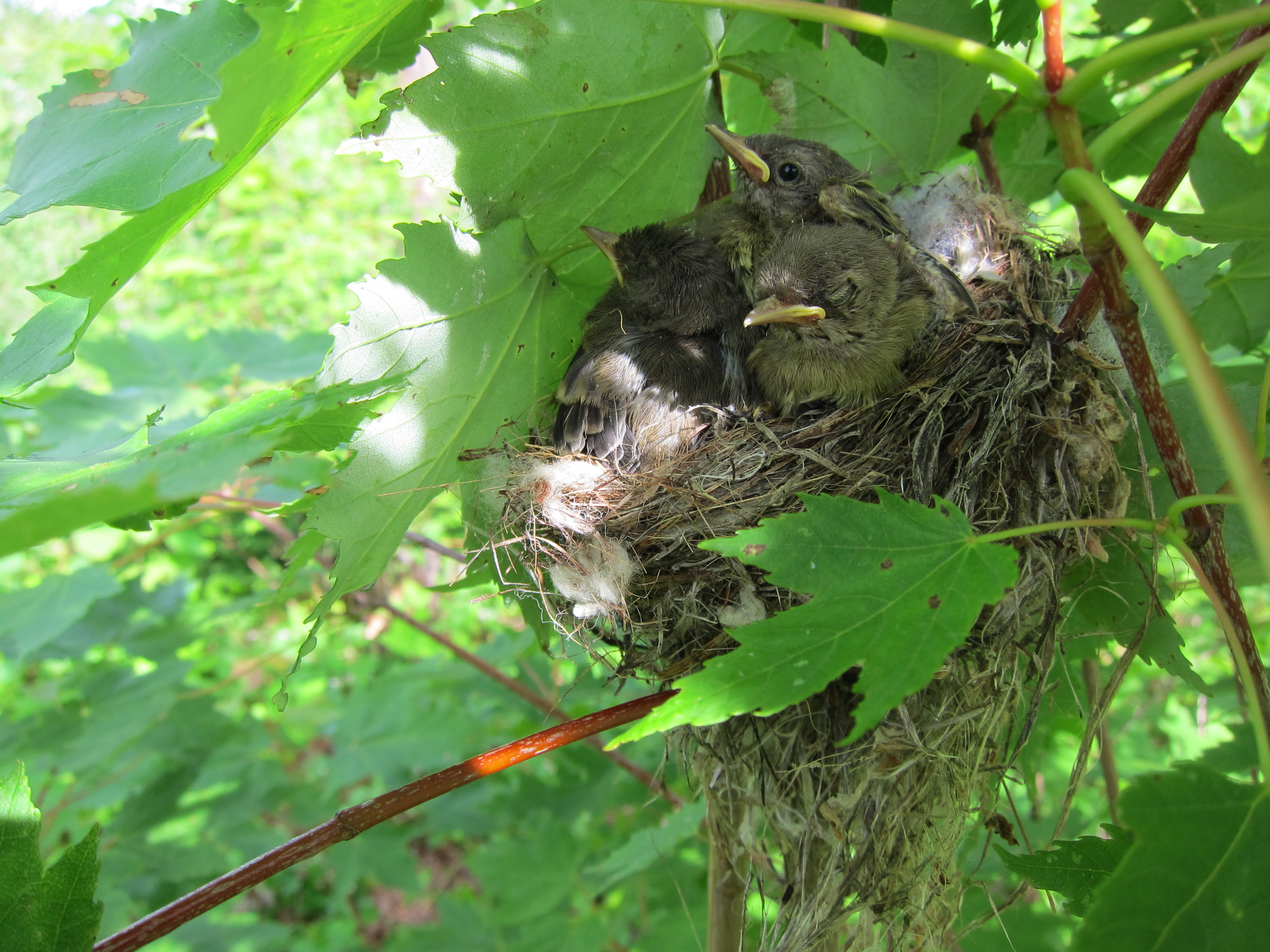

Supplement: Supplementary file 4 — Figure S4 [file ECE3-11-9241-s002.JPG]
