# Supplementary material for: Hatching date influences winter habitat occupancy: Examining seasonal interactions across the full annual cycle in a migratory songbird
Source: Ecol Evol. 2021 Jun 26;11(14):9241–53. doi: 10.1002/ece3.7500 (PMC8293775; doi:10.1002/ece3.7500)
Supplement: Supplementary file 5 — Appendix S1 [file ECE3-11-9241-s003.docx]

Appendix:

Hatching date influences winter habitat occupancy: examining seasonal interactions across the full annual cycle in a migratory songbird

Supplemental Figure 1

δ^13^C values in claws sampled from 13 individual prairie warblers that were initially captured and sampled upon arrival to the breeding grounds in Montague, MA and then recaptured and re-sampled during the same breeding season (on average 16 days later, range = 10-29 days, 69% of individuals were re-sampled 13-17 days after the initial capture). Points that fall close to the diagonal line (intercept = 0, slope = 1), represent similar δ^13^C values between the initial and recapture sample from the same individual.

Supplemental Figure 2

δ^13^C values in prairie warbler claws as a function of nestling body condition on the breeding grounds. Relationships shown separately for juvenile males and females within dry or wet winters. All relationships were not significant (*p >* 0.05).

Supplemental Figure 3.

Relationship between δ^13^C values in claws of adult male prairie warblers and fledging success of these males during the previous breeding season. Data were examined separately for wet and dry winters, and results were not significant (*p >* 0.05). Violin plots are shown, with points representing means and error bars as ± 1 SE.

Supplemental Figure 4.


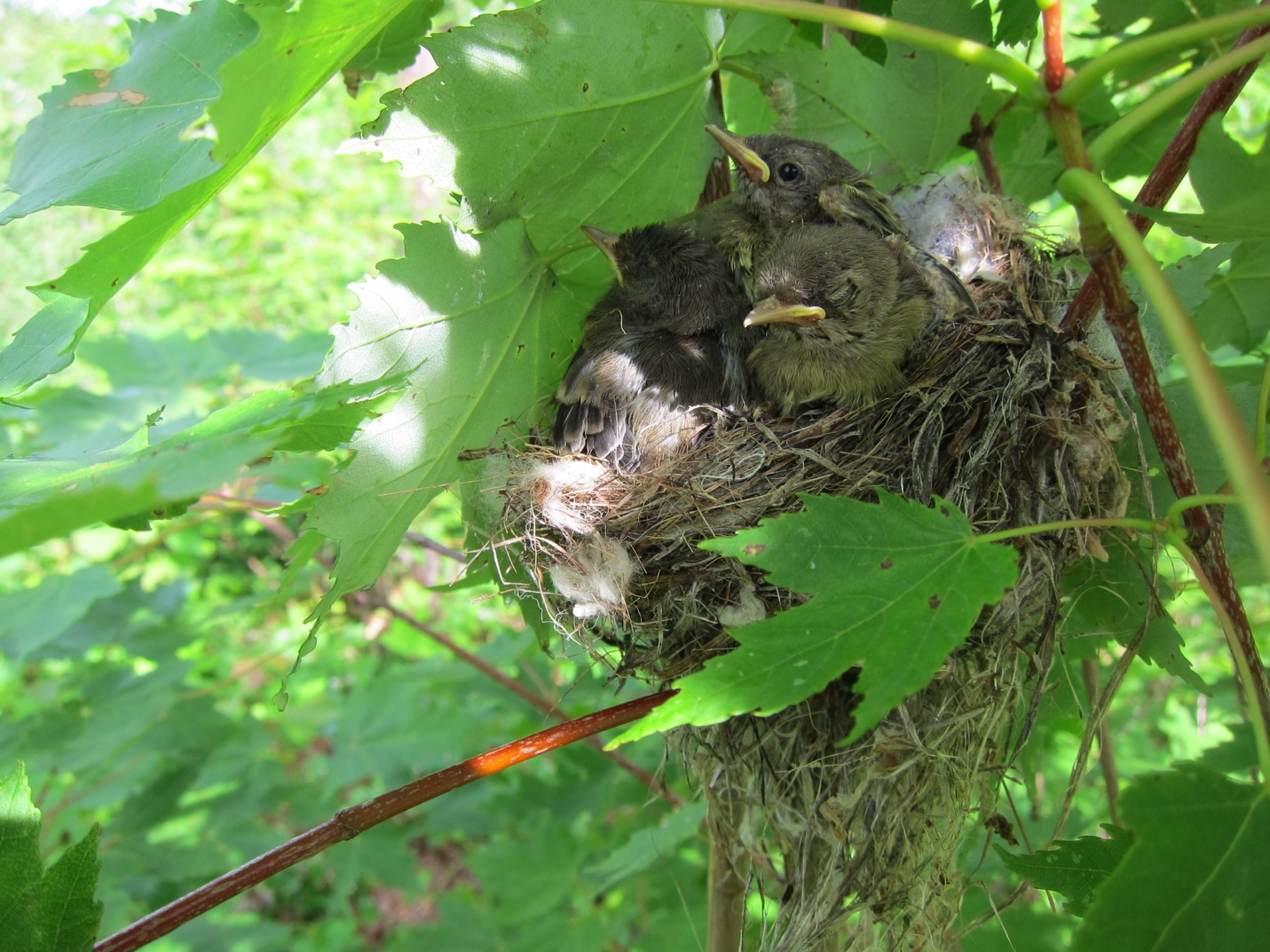


Prairie Warbler nestlings almost ready to fledge, in Montague, Massachusetts, USA.

Supplementary Table 1: Model parameter estimates, SEs, *t*-values, and *p*-values from the models conducted examining carry-over effects in prairie warblers. Random or fixed effects of year and/or individual were also included, depending on the model (see Methods and Results for more information). *n* = sample size.

| Subset | *n* | Response variable | Predictor variable | Estimate | SE | *t* | *p* | |
| --- | --- | --- | --- | --- | --- | --- | --- | --- |
| Nestling males, all years | 24 | δ^13^C values in claws | Intercept | -21.95 | 0.21 |  |  | |
|  |  |  | Hatch Date | 0.024 | 0.009 | 2.49 | 0.02 | |
|  |  |  |  |  |  |  |  | |
| Nestling females, all years | 13 | δ^13^C values in claws | Intercept | -21.53 | 0.31 |  |  | |
|  |  |  | Hatch Date | -0.0094 | 0.015 | -0.6 | 0.56 | |
|  |  |  |  |  |  |  |  | |
| Nestling males and females, dry winters | 25 | δ^13^C values in claws | Intercept | -22.19 | 0.22 |  |  | |
|  |  |  | Hatch Date | 0.035 | 0.012 | 2.9 | 0.008 | |
|  |  |  |  |  |  |  |  | |
| Nestling males and females, wet winter | 12 | δ^13^C values in claws | Intercept | -21.2 | 0.29 |  |  | |
|  |  |  | Hatch Date | -0.007 | 0.011 | -0.66 | 0.52 | |
|  |  |  |  |  |  |  |  | |
| Nestling males, dry winters | 18 | δ^13^C values in claws | Intercept | -22.24 | 0.23 |  |  | |
|  |  |  | Hatch Date | 0.04 | 0.012 | 3.27 | 0.005 | |
|  |  |  |  |  |  |  |  | |
| Nestling males, wet winter | 6 | δ^13^C values in claws | Intercept | -21.18 | 0.53 |  |  | |
|  |  |  | Hatch Date | -0.0026 | 0.017 | -0.15 | 0.89 | |
|  |  |  |  |  |  |  |  | |
| Nestling females, dry winters | 7 | δ^13^C values in claws | Intercept | -21.93 | 0.56 |  |  | |
|  |  |  | Hatch Date | 0.0097 | 0.036 | 0.27 | 0.80 | |
|  |  |  |  |  |  |  |  | |
| Nestling females, wet winter | 6 | δ^13^C values in claws | Intercept | -21.09 | 0.32 |  |  | |
|  |  |  | Hatch Date | -0.02 | 0.014 | -1.42 | 0.23 | |
|  |  |  |  |  |  |  |  | |
| Nestling males, all years | 24 | δ^13^C values in claws | Intercept | -21.49 | 0.11 |  |  | |
|  |  |  | Condition | -0.41 | 0.23 | -1.74 | 0.1 | |
|  |  |  |  |  |  |  |  | |
| Nestling females, all years | 13 | δ^13^C values in claws | Intercept | -21.65 | 0.15 |  |  | |
|  |  |  | Condition | -0.28 | 0.37 | -0.76 | 0.47 | |
|  |  |  |  |  |  |  |  | |
| Nestling males and females, dry winters | 25 | δ^13^C values in claws | Intercept | -21.6 | 0.12 |  |  | |
|  |  |  | Condition | -0.37 | 0.22 | -1.65 | 0.11 | |
|  |  |  |  |  |  |  |  | |
| Nestling males and females, wet winter | 12 | δ^13^C values in claws | Intercept | -21.34 | 0.17 |  |  | |
|  |  |  | Condition | 0.12 | 0.52 | 0.24 | 0.82 | |
|  |  |  |  |  |  |  |  | |
| Nestling males, dry winters | 18 | δ^13^C values in claws | Intercept | -21.55 | 0.13 |  |  | |
|  |  |  | Condition | -0.41 | 0.25 | -1.66 | 0.12 | |
|  |  |  |  |  |  |  |  | |
| Nestling males, wet winter | 6 | δ^13^C values in claws | Intercept | -21.21 | 0.26 |  |  | |
|  |  |  | Condition | 0.26 | 0.93 | 0.28 | 0.79 | |
|  |  |  |  |  |  |  |  | |
| Nestling females, dry winters | 7 | δ^13^C values in claws | Intercept | -21.77 | 0.28 |  |  | |
|  |  |  | Condition | -0.13 | 0.6 | -0.22 | 0.84 | |
|  |  |  |  |  |  |  |  | |
| Nestling females, wet winter | 6 | δ^13^C values in claws | Intercept | -21.51 | 0.26 |  |  | |
|  |  |  | Condition | -0.12 | 0.71 | -0.16 | 0.88 | |
|  |  |  |  |  |  |  |  | |
|  |  |  |  |  |  |  |  | |
| Adult males, drier winters | 55 | δ^13^C values in claws | Intercept | -21.91 | 0.18 |  |  | |
|  |  |  | Reproductive Success | -0.11 | 0.15 | -0.70 | 0.49 | |
|  |  |  |  |  |  |  |  | |
| Adult males, wet winter | 17 | δ^13^C values in claws | Intercept | -21.49 | 0.22 |  |  | |
|  |  |  | Reproductive Success | -0.38 | 0.35 | -1.1 | 0.29 | |
|  |  |  |  |  |  |  |  | |
| Adult males, drier winters | 30 | δ^13^C values in claws | Intercept | -22.01 | 0.28 |  |  | |
|  |  |  | Hatch Date of Young | -0.004 | 0.016 | -0.29 | 0.78 | |
|  |  |  |  |  |  |  |  | |
| Adult males, wet winter | 7 | δ^13^C values in claws | Intercept | -22.41 | 0.3 |  |  | |
|  |  |  | Hatch Date of Young | 0.044 | 0.019 | 2.28 | 0.07 | |
|  |  |  |  |  |  |  |  | |
| Adult males, all years | 44 | Hatch Date of Young | Intercept | 113.28 | 69.63 |  |  | |
|  |  |  | δ^13^C values in claws | 1.44 | 3.19 | 0.45 | 0.65 | |
|  |  |  |  |  |  |  |  | |
| ASY males, all years | 35 | Hatch Date of Young | Intercept | 134.03 | 85.41 |  |  | |
|  |  |  | δ^13^C values in claws | 2.38 | 3.9 | 0.61 | 0.55 | |
|  |  |  |  |  |  |  |  | |
| SY males, all years | 9 | Hatch Date of Young | Intercept | 52.73 | 129.22 |  |  | |
|  |  |  | δ^13^C values in claws | -1.33 | 5.99 | -0.22 | 0.83 | |
|  |  |  |  |  |  |  |  | |
| Adult males, drier winters | 30 | Hatch Date of Young | Intercept | 138.72 | 86.1 |  |  | |
|  |  |  | δ^13^C values in claws | 2.66 | 3.93 | 0.68 | 0.51 | |
|  |  |  |  |  |  |  |  | |
| Adult males, wet winter | 14 | Hatch Date of Young | Intercept | -12.01 | 127.28 |  |  | |
|  |  |  | δ^13^C values in claws | -4.48 | 5.88 | -0.76 | 0.46 | |
|  |  |  |  |  |  |  |  | |
| Adult males, all years | 112 | Hatch Date of Young | Intercept | 67.76 | 8.4 |  |  | |
|  |  |  | Arrival date | 0.36 | 0.23 | 1.59 | 0.12 | |
|  |  |  |  |  |  |  |  | |
| ASY males, all years | 90 | Hatch Date of Young | Intercept | 65.63 | 10.32 |  |  | |
|  |  |  | Arrival date | 0.43 | 0.29 | 1.49 | 0.14 | |
|  |  |  |  |  |  |  | |  |
| SY males, all years | 22 | Hatch Date of Young | Intercept | 64.87 | 17.02 |  |  | |
|  |  |  | Arrival date | 0.38 | 0.41 | 0.93 | 0.37 | |
|  |  |  |  |  |  |  |  | |
